# Numerical Modeling and Experimental Behavior of Closed-Cell Aluminum Foam Fabricated by the Gas Blowing Method under Compressive Loading

**DOI:** 10.3390/ma12101582

**Published:** 2019-05-15

**Authors:** Varun Sharma, Fatima Zivic, Nenad Grujovic, Norbert Babcsan, Judith Babcsan

**Affiliations:** 1 Faculty of Engineering, University of Kragujevac, Kragujevac 34000, Serbia; Varun.eu@gmail.com (V.S.); gruja@kg.ac.rs (N.G.); 2 ALUINVENT, Felsőzsolcai Industrial Park, Miskolc H-3561, Hungary; norbert@babcsan.hu; 3 University of Miskolc, Miskolc H-3561, Hungary; 4 Innobay Hungary Ltd., Miskolc 3519, Hungary; judit@babcsan.hu

**Keywords:** aluminum foam, closed-cell foam, micro-CT, tomography, modeling, mechanical properties

## Abstract

This paper deals with the experimental and numerical study of closed-cell aluminum-based foam under compressive loading. Experimental samples were produced by the gas blowing method. Foam samples had an average cell size of around 1 mm, with sizes in the range 0.5–5 mm, and foam density of 0.6 g/cm^3^. Foam samples were subjected to a uniaxial compression test, at a displacement rate of 0.001 mm/s. Load and stress were monitored as the functions of extension and strain, respectively. For numerical modeling, CT scan images of experimental samples were used to create a volume model. Solid 3D quadratic tetrahedron mesh with TETRA 10-node elements was applied, with isotropic material behavior. A nonlinear static test with an elasto-plastic model was used in the numerical simulation, with von Mises criteria, and strain was kept below 10% by the software. Uniform compressive loading was set up over the top sample surface, in the y-axis direction only. Experimental tests showed that a 90 kN load produced complete failure of the sample, and three zones were exhibited: an elastic region, a rather uniform plateau region (around 23 MPa) and a densification region that started around 35 MPa. Yielding, or collapse stress, was achieved around 20 MPa. The densification region and a rapid rise in stress began at around 52% of sample deformation. The numerical model showed both compressive and tensile stresses within the complex stress field, indicating that shear also had a prominent role. Mainly compressive stresses were exhibited in the zones of the larger cells, whereas tensile stresses occurred in zones with an increased number of small cells and thin cell walls.

## 1. Introduction 

Closed-cell Al-based foams belong to a group of cellular materials offering a wide choice in their design depending on the final product [1]. Their applications are versatile and significant attention has been given to the improvement of their properties, especially in structural applications such as energy absorbers in automotive and rail engineering [2]. Production technologies of these foams, aiming to control cell size and distribution within the material structure, have been investigated [2,3]. Different properties of these foams are important and those under study include: compressive mechanical properties and energy absorption [4], multifunctional applications [5], acoustic damping and sound absorption [6], electromagnetic shielding [7], and thermal conductivity [8]. The energy absorption property of aluminum-based foams has been used for different applications and represents a very important research area [9]; this is especially so for elements subjected to blast and impact [10,11,12], since these foams can efficiently mitigate shock, and absorb the energy of impulse loads. Aluminum foam has even been studied for use in nuclear transportation due to its energy absorption capabilities [13]. Mechanical behavior under compressive loads has been studied as the most probable loading mode for these foams [14,15,16], as well as at low strain regimes for high porosity foams [17]. Modeling of closed-cell foams, related to dynamic and static loading, is important for understanding and predicting the behavior of these structures [18,19]. Strain hardening has been suggested to initiate anisotropy, during uniaxial compression behavior [20,21]. Studies showed improvement in foam strength and elastic properties with an increase in relative density, under quasi-static compression [22,23]. The deformation mechanism of closed-cell Al-based foams largely depends on several influential factors, such as relative density, cell size and shape, cell wall thickness and void distribution, as well as parameters and types of production routes [1,24,25,26]. Young modulus and shear modulus were found to be inversely proportional to the cell size and cell wall variations [26].

Validated models showed that bending, shear, compressive and tensile stresses acting upon cells, govern deformation of the structure [27,28]. The correlation between structural parameters and compressive behavior has been studied to enable optimal design in relation to the elastic zone, yielding and collapse, the plateau zone and densification regimes [28,29]. The failure behavior of aluminum foams is under study and related numerical models have been investigated [29]. Computational homogenization of the structure, using analytical models with idealized geometry and isotropic properties, were applied to study possibilities of predicting the effective elastic properties of closed-cell aluminum foams [30]. The influence of the representative volume properties on mechanical behavior in finite element analysis is significant. Kovacik et al. [31] showed dependence of the Poisson ratio on foam density. This is important since all finite element modeling includes the value of the Poisson ratio as a basic material property and any changes to this affect the final numerical results. 

Geometry modeling (cell shape, meshing) has a governing influence on the process of finite element modeling (FEM) and analysis (FEA). There are numerous approaches to generate optimised geometry and representative volume, aiming to closely resemble the real structure, but also to consider computer resources and computational time [32,33,34,35,36,37]. Establishment of the relationship between changes at the micro scale and their influence on macro level properties is important, since it can enable suitable tailoring of the physical characteristics of foam. Variation in cell shapes usually results in changes of material behavior at the macroscopic level, due to different local deformation and failure [32,33]. Large variations in cell sizes and wall thicknesses are not favorable for good energy absorption [34]. The strain rate also has an influence on deformation mechanisms, accompanied by a strain-hardening effect, but it is a less influential parameter than cell wall thickness [35,36]. 

Computed tomography (CT) is powerful technique for characterization of real 3D structures, capable of producing high-resolution 3D structural images with nano- and micro- level details. Geometry modeling using CT scan images has been employed in FEA [38,39,40,41] in order to study the influences of microscopic structural properties (cell anisotropy and strut geometry) on macroscopic behavior. However, optimized selection of representative images, as well as the level of detail in computer meshing, is still the topic of investigation, in order to lower the computer resources and computational time that remain rather demanding for such modeling approaches. High-resolution images can be converted to meshes with extremely large numbers of nodes, thus resulting in overly demanding finite element processing.

This paper deals with finite element modeling (FEM) of closed-cell aluminum-based foam under compressive load, based on CT scan images extracted from a real experimental sample. Closed-cell foam was fabricated by the gas blowing method and experimental samples were subjected to compressive loading. Numerical results were compared with experimentally obtained stress–strain curves and analyzed. 

## 2. Materials and Methods 

**Figure 1:**
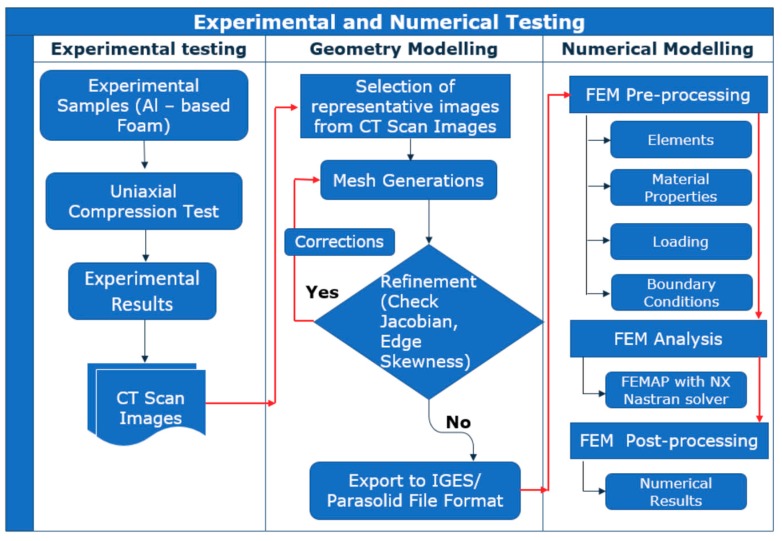
Schematic representation of steps in experimental and numerical study.

Our study realized experimental and numerical investigations, as shown in Figure 1. Experimentally prepared samples of closed-cell aluminum foam were subjected to compressive loading and the relationship between stress and strain was observed. Numerical modeling and simulation of the compressive loading of the closed-cell aluminum foam were performed using computed tomography (CT) scans obtained from experimentally prepared samples. CT scan images can provide high accuracy and enable 3D digitization of the physical model for computer-aided design (CAD) modeling and further creation of the volume model to be used in the numerical simulations and modeling [40]. 

### 2.1. Experimental Procedure

Closed-cell aluminum foam (Aluhab foam) was fabricated using the gas blowing method, at Aluinvent, Hungary. Aluhab is the Aluinvent trade name for aluminum foam manufactured from a special foamable aluminum alloy containing ultrafine sapphire particles, an Aluinvent proprietary technology [2,42]. Gas is blown into the melt with foamable liquid, through a small orifice, and bubble size can be adjusted by variation of process parameters. The diameter of the gas bubbles is around 1 mm according to the nozzle diameter. The average cell size of the closed-cell aluminum foam is around 1.0 mm, with cell sizes ranging from 0.5 mm up to 5.0 mm, and foam density of 0.6 g/cm^3^. Closed-cell aluminum foam samples were cut from the large block of Aluhab foam, using a bandsaw. There was no further post-processing of the sample prior to experimental testing.

**Figure 2:**
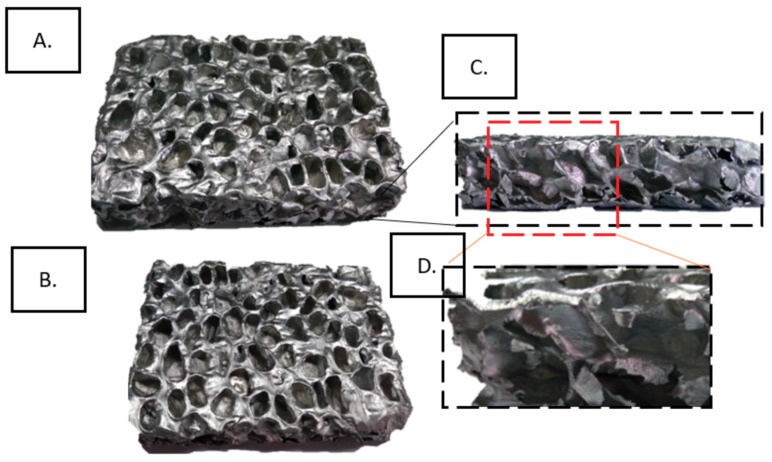
3D tomography images of the closed-cell aluminum foam samples (Aluhab foam): (**A**) front view; (**B**) back view; (**C**) side view; (**D**) enlarged view showing the cell arrangement.

**Figure 3:**
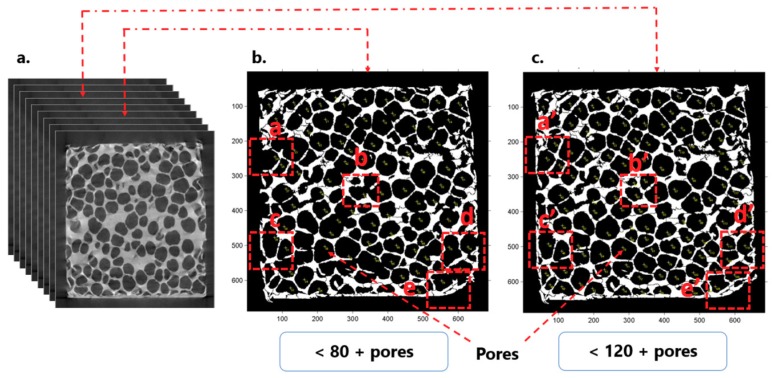
2D tomography images of the closed-cell aluminum foam samples (Aluhab foam): (**a**) CT scan images; (**b**,**c**) random position images, selected from the range of images generated during CT scanning, showing the differences in pore sizes and arrangements, and cell wall thicknesses throughout the 3D volume of the foam sample.

Characterization of the samples was realized using micro-CT, as described in [42]. A Hamamatsu L8121-03 Microfocus X-ray source (Hamamatsu Photonics, Hamamatsu, Japan) with the X-Ray tube having a focal spot size of 50 μm was used, with a maximum tube voltage of 150 kV. A Newport URS75 rotation stage (Newport Corporation, Irvine, USA) and Hamamatsu C7942SK-25 high-resolution imaging detector (Hamamatsu Photonics, Hamamatsu, Japan) were used, with 2000 projections per 360° rotation of the sample. 3D tomography images of the closed-cell aluminum foam samples are shown in Figure 2. Computed tomography (CT scan) 2D images are shown in Figure 3.

The uniaxial compression test was performed at room temperature using the universal testing machine (Hegewald and Peschke) (Hegewald & Peschke, Meß- und Prüftechnik GmbH, Nossen, Germany), at a displacement rate of 0.001 mm/s, in accordance with the DIN50134 standard [43]. Three tests were performed on three samples cut from the large foam block and elastic zones, plateau stress and densification zones were observed. Continuous measurement of the load as a function of time and extension, and of compression stress as the function of extension and strain was undertaken. 

### 2.2. Preparation of Simulation Volume Model Using a Three-Dimensional (3D) Digitization Technique

**Figure 4:**
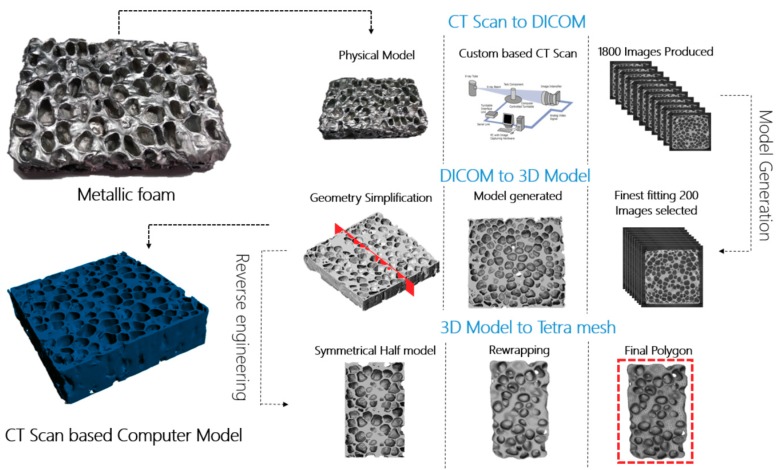
Creation of volume model based on CT scan images. CT scan images generated from the physical model were processed to create the sample for simulation and numerical modeling: CT scan-based computer model.

The volume model was created using CT scan images of experimentally prepared foam samples. A schematic representation of the preparation steps of the simulation model sample is given in Figure 4. 

**Figure 5:**
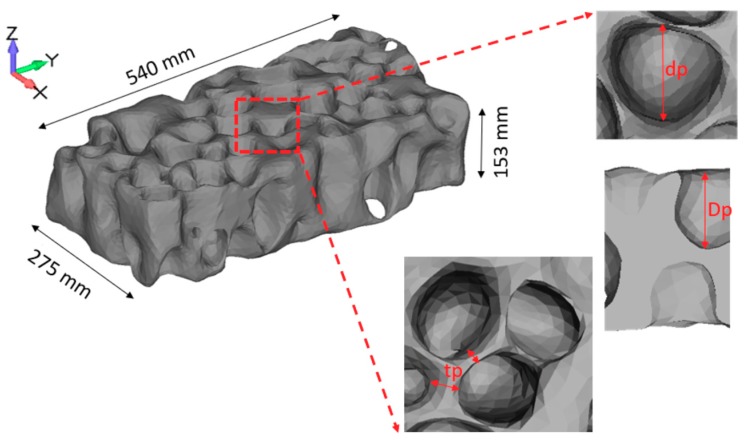
Numerical sample of the closed-cell foam based on CT scan images (Parasolid file format).

CT scan images have high accuracy, thus a very large number of points in the numerical point cloud can be generated that would result in accordingly very high demands on computer time and resources. Around 1800 CT scan DICOM (Digital Imaging and Communications in Medicine) images, were generated from the physical sample. For numerical modeling, we selected 200 DICOM images to represent the volume model of the foam; the differences between specific CT scan images can be seen in Figure 3. Images selected for the creation of the volume model should enable its close resemblance to the physical model. Appropriate selection of representative CT scan images is important for numerical description of the physical model and creation of the volume model, as elaborated in [42]. Selection of images was made using free open-source ImageJ CellProfiler software (Carpenter Lab, Broad Institute of Harvard and MIT, Cambridge, USA). The segmentation process started with the conversion of the original DICOM file format of the CT scan images to the STL, “stereolithography” file format, followed by adjusting the pixel values and optimal meshing in terms of calculation time and computer resources, as shown in Figure 4. The final numerical sample of the closed-cell foam is shown in Figure 5 and its geometrical properties are given in Table 1. For the representative volume, the volume model was cut into two symmetrical parts, as shown in Figure 4, in order to save computer time and resources. The numerical sample had more than 10 cells (Table 1) in order to ensure a sufficient number of cells to represent isotropic behavior and to consider the sample as a continuum, according to recommendations [2,42].

### 2.3. Numerical Modeling of Closed-Cell Aluminum Foam

Parabolic solid tetra elements (135843) were assigned to the geometry, with a global mesh size of 0.02 in the Cartesian coordinate system. Solid 3D quadratic tetrahedron mesh with TETRA 10-node elements was applied, with isotropic material behavior. The material properties of aluminum used in the finite element analysis, were as given in Table 2. In the case of metallic foam, Poisson’s Ratio has been observed to decrease with the decrease of foam density [31]. In the case of our foam sample, we concluded that a Poisson’s ratio of 0.3 was an acceptable value according to previous results [31]. Numerical calculations and finite element analysis were performed using Femap with NxNastran software (Version 10.3) (Siemens, Plano, USA). Open source MeshLab software (Version 2016.12) (Visual Computing Lab, Pisa, Italy) was used for geometry cleanup, refinement and meshing. 

A nonlinear static test with an elasto-plastic model was used in numerical simulation, with von Mises criteria applied for yield stress and shear failure analysis according to the DIN 50134 standard [43]. The von Mises yield criterion was deployed due to the ductile nature of aluminum. The material is elastic until yielding point and related equations are given in [44]. This material model is valid for small strain and calculations were automatically stopped when the threshold value for longitudinal strain was reached to keep the strain below 10%.

**Figure 6:**
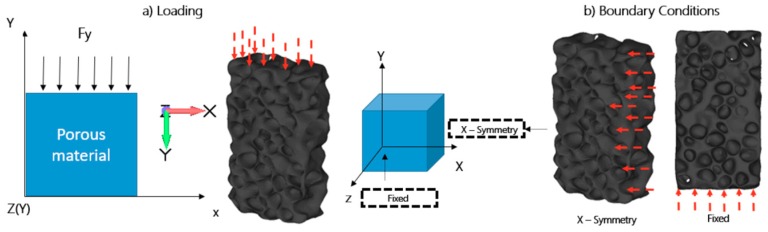
Schematic representation of: (**a**) loading direction and surface; (**b**) boundary conditions of the symmetrical model in finite element modeling.

Loading was applied on one side of the symmetrical model, comprising 129 nodes, as shown in Figure 6. Uniform compressive loading was set up over the top sample surface, in the y-axis direction only, with a fixed zx-plane. A maximum load of 50 kN was set up in numerical calculations, based on experimental results where a 90 kN load resulted in complete failure of the experimental sample. The full Newton–Raphson method was used for discretization of the partial differential equations and the iterative solution in the finite element analysis. Degrees of freedom are given in Table 3 for translation in the x-axis direction and rotation about the y and z axes.

## 3. Results and Discussion

### 3.1. Experimental Results

**Figure 7:**
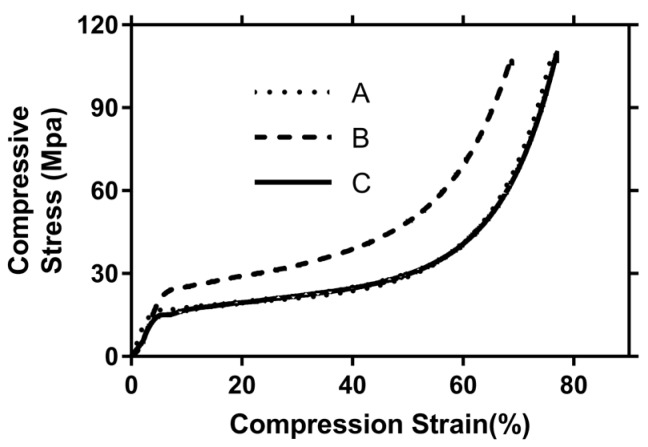
Compressive behavior of three foam samples (A, B, C) under uniaxial compression: Compression stress as the function of strain.

Three samples of Aluhab foam (A, B, C) cut from the same foam block were subjected to uniaxial compression. Compression stress as the function of strain is shown in Figure 7. It can be seen that one sample (B) exhibited different behavior. This is the consequence of the random position of A, B, and C samples within the same foam block, thus producing different sample surfaces upon which the loading acted. The distribution of cells over the surface was not the same for each of the A, B, and C samples due to different cutting lines. Also, the distribution of cells within the foam structure was not uniform, containing different cell sizes and diameters, thus resulting in small differences in the mechanical behavior of specific samples. Sample B comprised slightly more voids than samples A and C. Accordingly, the average of the stress and strain values was considered for further numerical calculations. Experimental tests showed that a 90 kN load produced complete failure of the sample. The level of stress during experimental uniaxial compression is given in Table 4. Three different zones can be observed during compression: an elastic region, a plateau region and a densification region. Yielding, or collapse stress, was achieved around 20 MPa. A rather uniform level of plateau stress, with very slightly increasing slope, occurred around 23 MPa, and densification started around 35 MPa. A densification region and rapid rise of stress began at around 52% of sample deformation, thus indicating acceptable agreement between the energy absorption behavior and results reported in the literature [4,14,41].

### 3.2. Numerical Modeling Results

**Figure 8:**
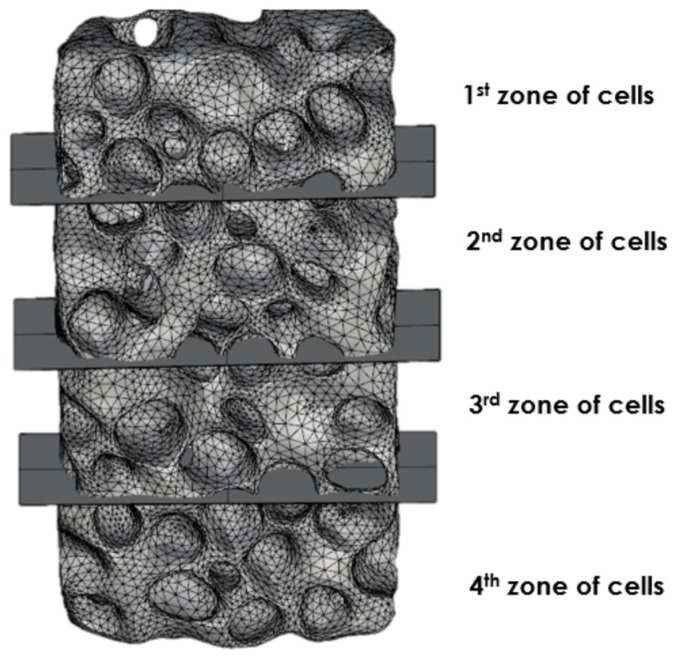
Partition of closed-cell numerical sample model into four zones for stress analysis throughout the volume model.

**Figure 9:**
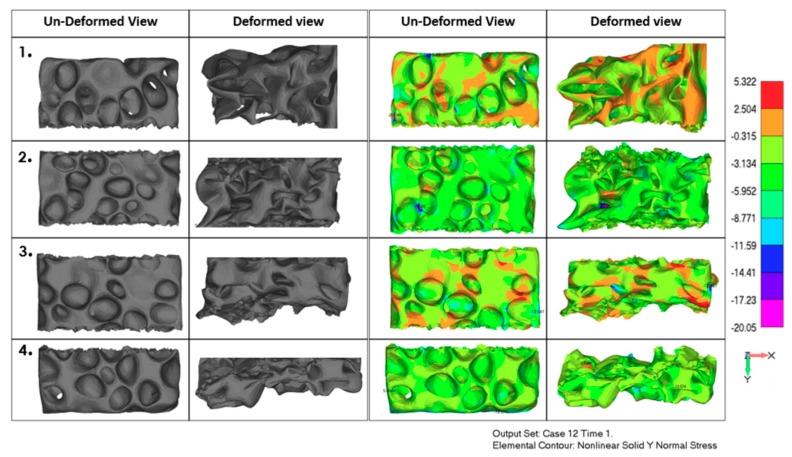
Numerical model in finite element analysis (FEA) in loading direction: Un-deformed view and deformed view. The two columns on the left show the geometrical model of the numerical sample; the two columns on the right show stress contour in loading (y-axis) direction.

Finite element modeling was performed, based on geometrical modeling using CT scan images of real experimental samples under compressive loading. The whole numerical sample that was subjected to the compressive test, was divided into four sections, in the direction of loading, as given in Figure 8. The nonlinear solid normal stress contour in the y-axis loading direction is given in Figure 9. Both compressive and tensile stresses can be observed in Figure 9, indicating that shear has a prominent role. As expected, the zones within the sample with the presence of larger voids, endured compression longer without complete crushing and densification, than in the case of zones containing smaller size cells. It can be seen that mainly compressive stresses were exhibited in zones of the larger cells, whereas tensile stresses occurred in zones with an increased number of small cells and thin cell walls. It can be observed that non-uniformity occurred in terms of stresses throughout the whole numerical sample. This implies that the uniform distribution of cells within the foam is very important, as well as uniform distribution of cell sizes, although these are difficult to control during the fabrication process. 

**Figure 10:**
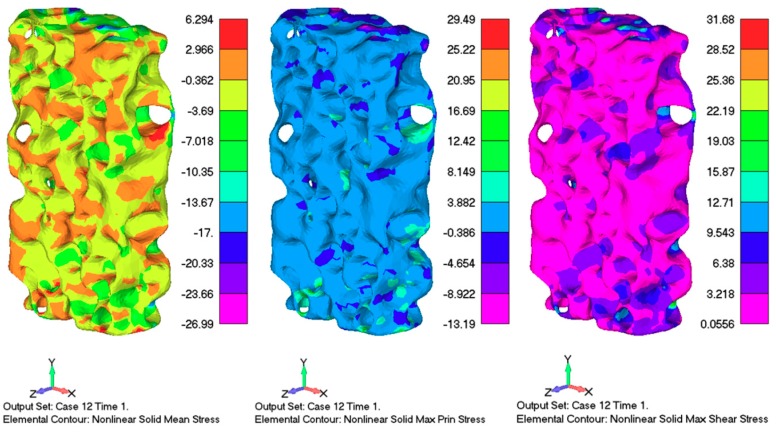
Stress contours of the numerical model under compressive load: Nonlinear solid mean stress (**left**); nonlinear solid maximum shear stress (**centre**); and nonlinear solid maximum principal stress (**right**).

As indicated in the literature, having cells of similar sizes uniformly distributed throughout the foam structure is hard to achieve, because the majority of large bubbles usually gather around the central zones, due to centrifugal force effects in the case of foam produced from molten solutions with foaming agents. However, compared to the literature data, there was rather good uniformity of void distribution throughout the Aluhab foam sample, as can be seen in the CT scan images in Figure 2 and Figure 3. This was also confirmed also by the experimental stress–strain curves in Figure 7, where a rather small difference in the behavior of only one sample (A) can be seen. From Figure 9 (far right column), it can be seen that tensile stresses occurred mainly within the sites of thin cell walls, whereas the closed cells positioned within thick bulk material mainly underwent compressive strain. It seems that material flow and shear is driven by topological heterogeneity and by spatial fluctuations of larger sized cells and their vicinity to each other. Linul et al. [45], showed that foam anisotropy has a significant influence on the compressive behavior of these closed-cell foams. They showed that, at room temperature, foam anisotropy had an effect on the yielding point, end of plateau region and start of the densification region [45]. Stress contours of the whole numerical model sample under compressive load are shown in Figure 10, indicating complex stress fields within the whole sample. It can be seen that higher levels of shear stress are related to the sites of thin cell walls, whereas thick solid material exhibited almost no shear. The stress contour of maximum principal stress indicated that large voids were subjected to the simultaneous actions of high compressive and tensile stresses, especially if these voids were positioned near the surface. Uniform solid material zones without larger voids, mainly exhibited moderate compressive stress. This is in accordance with findings proposed by Edwin Raj et al. [36] who elaborated that thin wall cells of aluminum foam tended to rapidly crack and collapse under loading, whereas thicker cell walls deformed with bending. 

**Figure 11:**
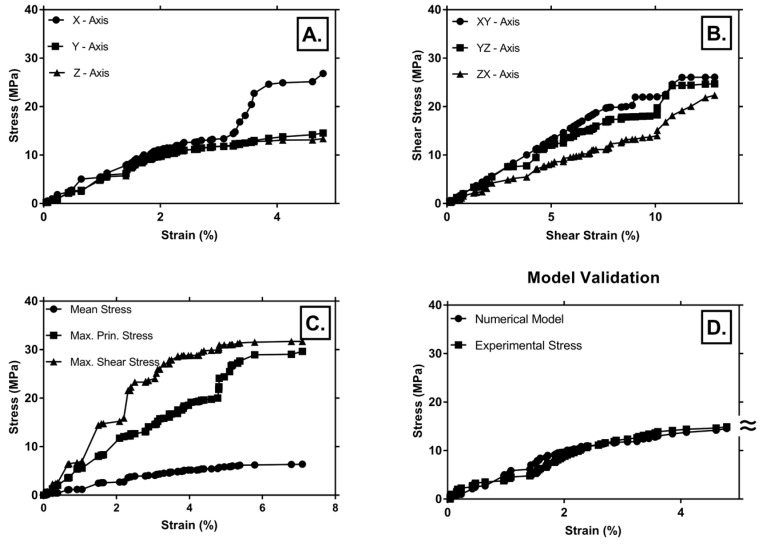
Stress–strain curves for numerical sample: (**A**) stresses in x-, y- and z-axis directions as a function of strain; (**B**) shear stresses in xy, yz and zx directions as a function of strain; (**C**) maximum principal stress, maximum shear stress, and maximum mean stress, as a function of strain; (**D**) comparison of stress–strain curves in the case of experimental and numerical tests.

Von Mises stresses in x, y, z directions, and along shear planes (xy, yz, zx), as well as the maximum principal stress, maximum shear stress, and maximum mean stress, as a function of strain, are given in Figure 11 (A-C). Comparisons of results obtained during experimental study and those from numerical modeling are shown in Figure 11D. From Figure 11C, it can be seen that collapse stress occurred around 5% strain and plateau stress started from 7% strain, in accordance with experimental results as given in Figure 7 and Table 4. Modeling was carried out under the limitation of a small strain (< 10%), thus densification strain was not achieved for the numerical sample. Modeling of the densification region would require a different simulation model, as shown by the authors of [14]. From Figure 11A,B, it can be observed that shear had a prominent role in the deformation of the sample, and was especially pronounced along the xy and yz planes. Longitudinal stress in the x-axis direction exhibited rapid increase of the stress for 3.5%–5% strain, if compared to y- and z-axis stress (Figure 11A). This indicated the increased presence of voids and thin structures (struts) in the x-direction, thus enabling their easy crushing and collapse. This is in accordance with other authors [14,36], who showed that collapse stress and plateau stress both have strong dependence on strain rate sensitivity. Thin walls and voids decrease overall sample strength and promote localized crushing and collapse of those cells. The stress–strain curves in Figure 11C are jagged, and especially pronounced ups and downs can be seen for shear stress and maximum principal stress, indicating sudden brittle fracture of the cell walls. This is also confirmed by other authors [46], who showed that under compression, high porosity resulted in serrated stress–strain curves with local peaks, due to stress release after the localized fracture of cell walls, whereas low porosity aluminum foams exhibited smooth curves. They also showed that increase of porosity resulted in decrease of the yield strength [46]. Our model validation showed acceptable agreement between experimental and numerical results as shown in Figure 11D, within the linear elastic region.

## 4. Conclusions

Numerical modeling of a uniaxial compression test of Aluhab foam was performed based on CT scan images produced from experimental tests. Modeling was carried out under the limitation of a small strain (< 10%), thus densification strain was not achieved for the numerical sample. Complete failure of the sample was experimentally achieved at a 90 kN load, and accordingly 50 kN was adopted as the maximum load in the numerical study. A rather linear elastic region (up to 17 MPa) and uniform plateau region (around 23 MPa) were exhibited, whereas densification started at around 52% of sample deformation. There was rather good uniformity of void distribution throughout the sample. Tensile stresses occurred mainly within the sites of thin cell walls, whereas the closed cells positioned within thick bulk material mainly underwent compressive strain, altogether indicating complex stress fields within the whole sample, with shear having a prominent role. Higher levels of shear stress were related to the sites of thin cell walls, whereas thick solid material exhibited almost no shear. Large voids were subjected to the simultaneous actions of high compressive and tensile stresses. Numerical results showed jagged stress–strain curves indicating sudden brittle fracture of the cell walls. It seemed that material flow and shear were driven by topological heterogeneity and by spatial fluctuations of larger sized cells and their vicinity to each other. The numerical and experimental results indicated that collapse stress occurred at around 5% strain and plateau stress started from 7% strain. Within the linear elastic region, our model showed acceptable agreement between experimental and numerical results. Modeling related to the densification region would require a different simulation model. 

## Figures and Tables

**Table 1:** Properties of the numerical sample.

Total Cells	Cell Wall Thickness, *tp* (mm)	Radial Cell Diameter, *dp* (mm)	Cell Depth, *Dp* (mm)
38-42	9–12	50–60	45–50

**Table 2:** Material properties of aluminum [43].

Density (tonne/mm^3^)	Modulus of Elasticity (M Pa)	Yield Stress	Poisson Ratio
2.7 x 10^-9^	68200	55 MPa	0.3

**Table 3:** Degrees of freedom for modeling of closed-cell Aluhab foam.

Cartesian Coordinate Axes	Degree of Freedom					
	Translation in *x* Direction	Translation in *y* Direction	Translation in *z* Direction	Rotation in *x* Direction	Rotation in *y* Direction	Rotation in *z* Direction
*x*–symmetry	✓				✓	✓
Fixed Node	✓	✓	✓	✓	✓	✓

**Table 4:** Stress levels for specific regimes and densification strain during uniaxial compression of Aluhab foam.

	Linear Elastic Zone (Cell Wall Bend)	Plateau Zone – Collapse (Cell Wall Buckle, Yield or Fracture)	Densification Zone (Cell Wall Crush Together)	Densification Strain
Sample A	0–17 Mpa	17–31 MPa	31–104 MPa	55%
Sample B	0–23 Mpa	23–37 MPa	37–105 MPa	50%
Sample C	0–14 Mpa	14–32 MPa	32–107 MPa	56%
